# Assessing strategies to minimize unintended fitness consequences of aquaculture on wild populations

**DOI:** 10.1111/eva.12089

**Published:** 2013-10-09

**Authors:** Marissa L Baskett, Scott C Burgess, Robin S Waples

**Affiliations:** 1 Department of Environmental Science and Policy, University of CaliforniaDavis Davis, CA, USA; 2 Center for Population Biology, University of CaliforniaDavis Davis, CA, USA; 3 Northwest Fisheries Science Center, National Marine Fisheries Service, National Oceanic and Atmospheric AdministrationSeattle, WA, USA

**Keywords:** aquaculture, contemporary evolution, domestication selection, migration load, quantitative genetic model, *Salmo salar*

## Abstract

Artificial propagation programs focused on production, such as commercial aquaculture or forestry, entail strong domestication selection. Spillover from such programs can cause unintended fitness and demographic consequences for wild conspecifics. The range of possible management practices to minimize such consequences vary in their control of genetic and demographic processes. Here, we use a model of coupled genetic and demographic dynamics to evaluate alternative management approaches to minimizing unintended consequences of aquaculture escapees. We find that, if strong natural selection occurs between escape and reproduction, an extremely maladapted (i.e., nonlocal-origin, highly domesticated) stock could have fitness consequences analogous to a weakly diverged cultured stock; otherwise, wild population fitness declines with increasing maladaptation in the cultured stock. Reducing escapees through low-level leakage is more effective than reducing an analogous number of escapees from large, rare pulses. This result arises because low-level leakage leads to the continual lowering of wild population fitness and subsequent increased proportional contribution of maladapted cultured escapees to the total population. Increased sterilization efficacy can cause rapid, nonlinear reductions in unintended fitness consequences. Finally, sensitivity to the stage of escape indicates a need for improved monitoring data on how the number of escapees varies across life cycle stages.

## Introduction

Cultivation of organisms for production purposes such as agriculture, livestock, forestry, and aquaculture inevitably involves strong artificial selection, both intentional and unintentional, on a variety of morphological, physiological, behavioral, and life-history traits (Ledig 1992; Mignon-Grasteau et al. 2005; Hutchings and Fraser 2008). In many cases, cultivation programs occur in the same location as wild conspecifics, which can lead to large escape of cultured-origin individuals that interact and interbreed with wild populations. Artificial selection in cultivated environments means that such spillover can have unintended fitness consequences for wild populations (Laikre et al. 2010).

Commercial commodity aquaculture (i.e., full life cycle rearing with a goal of complete capture for production; Lorenzen et al. 2012; Utter and Epifanio 2002) exemplifies this potential for cultivation to affect the fitness of wild populations. Artificial selection in aquaculture environments on traits such as growth, maturation, disease resistance, feeding aggression, and predator avoidance behavior occurs in a variety of cultivated species, including sea bass, tilapia, catfish, Atlantic cod, and salmon (Youngson et al. 2001; Hutchings and Fraser 2008). In addition, many aquaculture programs involve growth of the cultured individuals in semi-closed facilities, such as net pens, that are located in the same freshwater, marine, or estuarine environment as conspecifics, such that spillover has the potential to affect the fitness and dynamics of wild populations (Youngson et al. 2001; Naylor et al. 2005; Hutchings and Fraser 2008; Jensen et al. 2010; Lorenzen et al. 2012).

Aquaculture escapees can lead to particularly strong fitness consequences for wild populations when the latter have low or depleted population sizes. For example, Atlantic salmon (*Salmo salar*) aquaculture occurs in many locations where local, conspecific wild populations are threatened or endangered (National Research Council 2004; Morris et al. 2008). Aquaculture escapees can make up a significant proportion of wild populations but vary dramatically across space and time (Gross 1998; Morris et al. 2008). Documented fitness consequences of escaped farmed salmon for wild populations include effects on survival, growth, maturation, and reproductive success (Gross 1998; McGinnity et al. 2003; Fraser et al. 2010). Aquaculture also can have a variety of nongenetic impacts on wild populations, such as direct competition and disease spread (Gross 1998; Naylor et al. 2005). Empirical investigation into the role of hybridization with conspecifics indicates a significant role for interbreeding and its fitness consequences in overall aquaculture effects (e.g., McGinnity et al. 2003; Skaala et al. 2012; synthesized in a meta-analysis by Ford and Myers 2008).

A number of management approaches can influence the fitness consequences of cultured escapees on wild populations. First, a reduction in the number of escapees (i.e., containment) directly reduces the demographic contribution to wild populations (Hindar et al. 1991; Gross 1998; Youngson et al. 2001). Spillover occurs through a variety of process, ranging from loss of entire net pens due to storms to leakage during harvesting, net-pen changes, and movement (Gross 1998; Youngson et al. 2001; Naylor et al. 2005; Thorstad et al. 2008; Jackson et al. 2012). Spillover can also occur at any life cycle stage, including eggs from net-pen spawning (Gross 1998; Youngson et al. 2001; Jensen et al. 2010; Uglem et al. 2012), and patterns and frequency of escape can depend heavily on species-specific behaviors (Jensen et al. 2010; Jackson et al. 2012). Therefore, the specific management approach to reducing spillover will affect the temporal pattern of spillover within and across life cycles.

Within the life cycle, management choices, such as retention in fully closed terrestrial facilities until later development stages (Hindar et al. 1991), changes to harvest equipment that affect leakage (Naylor et al. 2005), or harvest before substantial net-pen spawning occurs (Uglem et al. 2012), can differentially affect spillover at different life cycle stages. Escapes that occur at early life-history stages experience more natural processes that might reduce escapee survival before interbreeding and subsequent genetic effects occur (Waples et al. 2012). However, escapes that occur at early life-history stages will also have a greater duration of demographic interactions (e.g., competition) with wild-origin fish (Hindar et al. 1991). Therefore, assuming the goal is to minimize unintended consequences for wild populations, the relative efficacy of different strategies to reducing escapees at different life cycle stages will depend on the relative roles of demographic and genetic interactions in determining the effects of escapees as well as the timing of critical density-dependent interactions in the life cycle.

Across life cycles, how much of spillover occurs in rare, large pulses versus constant, low-level leakage will depend on choices such as net-pen reinforcement to reduce likelihood of loss during a storm, placement of net pens in more sheltered locations, or alterations to net-pen rotation and change practices (Gross 1998; Youngson et al. 2001; Naylor et al. 2005). One might logically expect that large pulses of escapees might be less frequent but have substantial fitness effects when they occur, while constant, low-level leakage might have smaller effects per spillover event but a more consistent accumulation of events over time. In reality, spillover occurs with high variability in time (Morris et al. 2008) that integrates across these extremes as well as a variety of intermediate cases. Therefore, the relative efficacy of different strategies to reducing escapees with different degrees of temporal variability will depend on the relative importance of the quantity of an individual spillover event and the cumulative effect of multiple such events.

Second, sterilization of aquaculture fish can reduce interbreeding with wild populations and therefore genetic effects (Hindar et al. 1991; Youngson et al. 2001; Naylor et al. 2005). However, escaped sterilized fish will still compete with wild fish (Hindar et al. 1991), and sterilization is often only partially effective so some interbreeding might occur, depending on the species (where sterilization programs occur in a variety of aquaculture species such as salmon, trout, catfish, carp, oysters, and mussels; Piferrer et al. 2009). Therefore, the potential for sterilization to have a significant effect on minimizing unintended consequences of escapees will depend on the relative roles of demographic and genetic interactions, especially the relative importance of the total amount of interbreeding that occurs.

Finally, the degree of maladaptation of the cultured population has a substantial influence on the consequences of escapes for wild population fitness (Fleming 1995; Tufto 2001). While fitness consequences typically increase with increasing maladaptation, eventually with extreme maladaptation, the escapees might be unlikely to survive or reproduce in the wild and therefore fitness consequences will decrease (Fleming 1995; Lorenzen 2005), depending on the order of events in the life cycle (in particular, the potential for natural selection to filter the maladapted cultured escapees before reproduction; Baskett and Waples 2013). In aquaculture, domestication selection for commercially desirable traits will always cause some degree of maladaptation (Hutchings and Fraser 2008; Lorenzen et al. 2012). However, that degree might depend on the duration in captivity and whether the aquaculture broodstock is of local or nonlocal origin (Hutchings and Fraser 2008; Lorenzen et al. 2012). The high degree of variation in ages of various aquaculture programs and regulations as to whether importation of nonlocal, highly domesticated stocks is permitted (e.g., O'Reilly et al. 2006) will mean a high degree of variation in the degree of maladaptation across programs. Therefore, a comprehensive evaluation of the fitness consequences of escapees requires an exploration across this range of variation in degree of maladaptation.

Here, we develop a quantitative framework to evaluate the consequences of (i) the degree of maladaptation in the cultured population, (ii) constant low-level spillover versus rare, large pulses of escapees, (iii) the amount of reduced reproductive success (sterilization efficacy) of cultured escapees in the wild, and (iv) spillover of different life-history stages. Expanding on a variety of existing models that demonstrate the potential for aquaculture escapees to have demographically relevant fitness effects on wild populations (e.g., Hutchings 1991; Fleming 1995; Tufto 2001, 2010; Hindar et al. 2006), we integrate the range of alternative management approaches to reducing such effects for a comprehensive, comparative evaluation of their relative efficacy. For this evaluation, we build on an existing framework that explores management approaches to reducing unintended fitness consequences of hatcheries (Baskett and Waples 2013). The hatchery model includes full intermixing of the captive-reared and wild populations after hatchery release and a dynamical hatchery population derived from this mixed population (i.e., two-way gene flow); in contrast, our aquaculture model here assumes a closed, constant cultured population from which spillover occurs to the dynamical wild population (i.e., one-way gene flow). Furthermore, while the management questions of the degree of maladaptation in the cultured population and the stage of release or spillover are common to both explorations, the questions of type of spillover (constant low-level leakage versus rare, large pulses) and (potentially imperfect) sterilization are unique to the aquaculture exploration here.

## The model

To maintain generality across a variety of cultured programs, we sought to construct the simplest possible model that incorporates the dynamics relevant to the above-described management comparisons and focus on qualitative trends. In the model detailed mathematically below, we follow the joint genetic and demographic dynamics of a wild population experiencing inputs from a (demographically and genetically) constant cultured population. For the genetic dynamics, because many of the traits under selection in cultured environments are quantitative traits, we use a quantitative genetic model of a generic trait and follow the full breeding value distribution. The difference between the mean of the cultured population distribution and the optimal trait in the wild (i.e., the trait value that maximizes fitness in the natural selection function; the wild trait distribution itself is dynamical) represents the degree to which the cultured individuals are maladapted to the wild environment. This generic approach might represent a number of traits under domestication selection that affect fitness in the wild, such as egg size, body size, predator avoidance, and territoriality (Reisenbichler and Rubin 1999; Hutchings and Fraser 2008).

**Figure 1:**
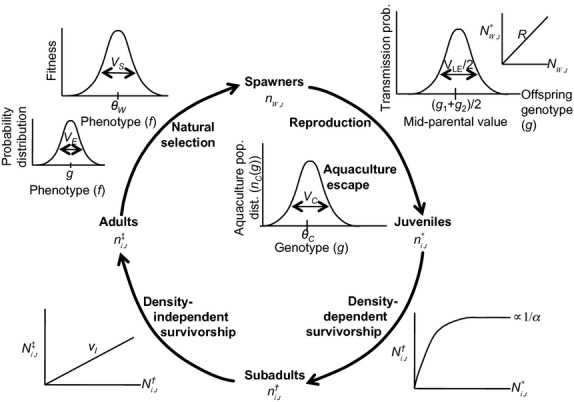
Model illustration given the default life cycle order and aquaculture escape timing (eqns 1–7). Graphs illustrate the dynamics at each step, with one full cycle for each generation (stages indicated on the circle, wild population dynamics outside the circle, and aquaculture population dynamics inside the circle). See Table 1 for definitions of the parameters indicated. Model explorations include changing the order of aquaculture escape, density-dependent survival, density-independent survival, and natural selection.

A single time step represents a generation, which includes five events (Fig. 1): reproduction, density-dependent survival, density-independent survival, natural selection, and escape of cultured individuals. We explore all possible orderings of the latter four events to evaluate the effect of the timing of escape relative to other life-history events. The spillover can occur with a constant proportion each generation, with a stochastically variable proportion each generation, or as a binary event of either all or none of the cultured individuals escaping with a given probability; in all cases we assume the same average number of escapees over multiple generations. At reproduction where surviving cultured escapees interbreed with the wild population, individuals of cultured origin can have lower reproductive success than individuals of wild origin; this models partial or full sterilization as well as any nongenetic effects of domestication on reproductive success.

## Mathematical model details

Here, we mathematically detail the model given the default life cycle ordering of reproduction – escape – density-dependent survival – density-independent survival – selection. We model the different life cycle orderings by rearranging the equations presented below, with only post-escape dynamics being applied to the population of cultured origin. Throughout, the model follows population density *n*_*i,t*_ (g) of genotypes *g* over time *t* for population of origin *i* (*i* = *W* for wild or *C* for cultured), where total population size is 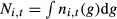 and the genotype probability density is *φ*_*i*,*t*_(*g*) = *n*_*i*,*t*_(*g*)/*N*_*i*,*t*_ (analogous to Coulson et al. 2010).

For reproduction in the wild environment, we use the infinitesimal model of quantitative genetics (Turelli and Barton 1994, based on the assumption that a large number of unlinked loci determine the genetic component of a phenotype). This model integrates the product of the probability that two individuals with genotypes *g*_1_ and *g*_2_ encounter each other and the probability distribution of offspring genotypes from such an encounter over all possible mating pairs. Assuming random mating, the encounter probability is the product of the frequency of each genotype *φ*_*W*,*t*_(*g*_1_)*φ*_*W*,*t*_(*g*_2_). The parent–offspring transmission function provides the probability distribution of offspring genotypes as normally distributed around the mean parental genotype with variance of half of the genetic variance at linkage equilibrium *V*_*LE*_, that is, 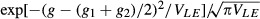. We translate the resulting offspring genotype probability density into the population density by multiplying by the total parental population size *N*_*W,t*_ and the average number of offspring per individual *R* to arrive at


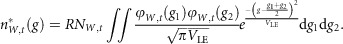
(1)

We also explore a model extension that includes assortative mating by the same phenotype under natural selection. We implement assortative mating as a phenotypic correlation between mating pairs (e.g., as might occur if the phenotype is body size or spawn time) with the same mathematical approach as Baskett and Waples (2013).

For escape of cultured individuals into the wild environment, we assume escapees come from a cultured population of size *N*_*C*_ with mean genotype *θ*_*C*_ and additive genetic variance *V*_*C*_. For each generation *t*, a proportion *p*_*t*_ of this population escapes to determine the population density of individuals of cultured origin


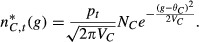
(2)

Because time is in units of generations, multiplying out annual spillover, as it is typically recorded, over a generation would be necessary to arrive at the relevant spillover numbers. We implement constant, variable, and pulsed escapes such that all have the same average escapee proportion over time *p*_*c*_. For constant spillover, *p*_*t*_ = *p*_*c*_ independent of time *t*. For pulsed spillover, the probability of an entire net-pen loss in a given generation (*p*_*t*_ = 1) is *p*_*c*_; otherwise, no spillover occur (*p*_*t*_ = 0), such that 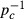 is the average number of generations between pulses. For variable spillover, in each generation the proportion of escapees is a random number drawn from the binomial distribution according to *p*_*t*_ ∼ Binom(*X*, *p*_*c*_)/*X*, where the number of Bernoulli trails *X* determines the variance in escapees. Under this implementation, the constant escape simulations represent low-level leakage, the pulsed escape simulations represent the rare case of loss of the entire net-pen facility during particularly large storms, and the variable escape simulations represent the combination of large and small leakages that might occur through a variety of processes such as net-pen tears, net-pen changes, and partial storm damage. In reality, all of these processes occur simultaneously, but we explore each individually to separate the relative effect of each.

We implement density dependence using the Beverton–Holt functional form given strength α, such that the post-density-dependent population distribution is


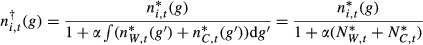
(3)

for both the captive-origin (*i* = *C*) and wild-origin (*i* = *W*) populations. If escape occurs after density dependence, then we exclude 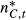 from this equation.

Density-independent survival occurs with probability ν_*I*_ to lead to a population distribution of


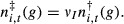
(4)

For natural selection, we first translate the genotype *g* into a phenotype *f* assuming random environmental effects, that is, an individual's phenotype is randomly distributed around its genotype with environmental variance *V*_*E*_ (no adaptive phenotypic plasticity). Then, natural selection acts on the phenotype, where we implement stabilizing selection for optimal trait *θ*_*W*_ with variance in the selection surface *V*_*S*_; the inverse of this variance represents the strength of selection. Therefore, the postselection population density is


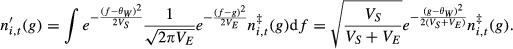
(5)

Given these parameters, the mean fitness of the wild spawner population is


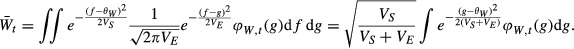
(6)

Before reproduction, we combine the wild-origin and cultured-origin populations to form the new wild spawner population. In the process, cultured-origin individuals have a potentially lower relative spawning success ν_*S*_, which might occur due to nongenetic effects of rearing in the captive environment that affects spawning success (e.g., ability to migrate to spawning grounds, developmental responses to the cultured environment; Gross 1998; Youngson et al. 2001; Lorenzen et al. 2012) and due to any sterilization. Applying this relative spawning success and combining the captive-origin and wild-origin populations, the wild spawner population in the next generation is


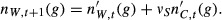
(7)

## Model implementation and analysis

We numerically analyze the above-described model by iterating through eqns 1–7 given the parameter values in Table 1. We chose these parameter values as generic values that result in a significant fitness and demographic impact of the cultured population on the wild in the default case, such that the further explorations can indicate how the different management choices and scenarios affect that impact. In addition, we explore the model sensitivity to all parameter values. Therefore, our simulations do not represent a specific species such as Atlantic salmon, but rather, we focus our conclusions on qualitative trends that are consistent across a range of parameter values and might apply broadly. To numerically implement eqn (1), we employ the Fourier transform method described in Turelli and Barton (1994).

**Table 1:** Parameters and default values. Unless otherwise indicated, the parameter values here apply to the results in Figs 8

Parameter	Default value	Description
*V*_*LE*_	0.01	Within-family genetic variance
*θ*_*W*_	1	Optimal trait in the wild
*V*_*S*_	0.1	Variance in selection surface, 1/selection strength
*V*_*E*_	0.01	Environmental variance
*R*	3	Reproductive output
α	0.0005	Beverton–Holt density-dependent parameter
ν_*I*_	0.75	Density-independent survival
ν_*S*_	0.8	Relative spawning success of aquaculture-origin fish
*θ*_*C*_	0–1	Aquaculture mean/optimal trait
*V*_*C*_	0.01	Genetic variance in the aquaculture population
*N*_*C*_	20 000	Captive population size
*p*_*c*_	0.05	Proportion of escapees/probably of full escape
*a*	0	Strength of assortative mating

We evaluate three central metrics that indicate the genetic and demographic effects of cultured escapees. First, we use equilibrium wild spawner population size 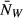 as an indicator of the demographic effects of the cultured population. Second, we use equilibrium wild spawner mean fitness 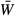 (eqn 6) as an indicator of the genetic effects of the cultured population. Third, we evaluate the time it takes a population, starting at the equilibrium for a given aquaculture scenario, to recover to 95% of the size it would be without aquaculture after cessation of cultured escapees (threshold chosen to reflect a population size that would be considered as recovered in a statistical test of empirical observations), which depends on the combined demographic and genetic effects of spillover. We find that these three metrics capture additional possible metrics relevant to the genetic and demographic effects of aquaculture spillover and its management. Specifically, fitness captures migration load as well as changes in the genetic mean and variance, population size reflects the fraction of natural spawners of natural origin, and post-aquaculture recovery time parallels the effect of a continued aquaculture program on the recovery of a small wild population (Supporting Information Appendix A).

We focus our analysis on equilibrium outcomes to allow qualitative comparison of the long-term effect of different management choices, as many aquaculture programs have no plans to stop and to avoid an arbitrary choice of culture program duration that will increase the sensitivity of our results to parameter values and model assumptions that affect the timescale of evolution. First, we initialize the model at the simulated equilibrium without aquaculture, and then, we run the model to equilibrium or, in stochastic runs, quasi-equilibrium. To ensure that the simulations have reached equilibrium, we choose a run time (150 time steps, or wild population generations) well past the equilibrium point for any combination of parameter values (e.g., the time to 50% equilibrium under the default parameter values is 31 time steps) and present the metrics at the last time step for deterministic simulations (constant spillover). For stochastic simulations with variable or pulsed spillover, we run the model for double the time and present the 1st, 25th, 50th, 75th, and 99th percentiles of the distribution of each metric for the second half of 50 independent simulations to capture the full distribution at quasi-equilibrium. We use both multiple time points within a time series and multiple runs to capture the distribution both as might be observed over time within an aquaculture program and across independent runs. The relative, qualitative trends that are the focus of our analysis do not change with the use of a shorter run time (Supporting Information Appendix A).

We explore the equilibrium metrics given different life cycle orderings and release timing under constant spillover. Then, for the default life cycle ordering (reproduction – escape – density dependence – density-independent survival – selection), we explore the effect of constant, variable, or pulsed spillover. Finally, we explore the effect of the relative cultured-origin spawning success parameter ν_*S*_ to determine the potential efficacy of partial (0 < ν_*S*_ < 1) or full (ν_*S*_ = 0) sterilization. The case of ν_*S*_ = 0, or no reproductive input from the cultured population, also allows us to isolate the demographic effect of cultured escapees and compare that with the combined demographic and genetic effect when ν_*S*_ > 0. In all cases, we explore how the outcome depends on the value of the mean aquaculture phenotype *θ*_*C*_, which indicates the degree of maladaptation in the aquaculture population: *θ*_*C*_ = 1 indicates that cultured individuals are optimally adapted to the wild on average, and the degree of maladaptation increases as *θ*_*C*_ decreases. Although achieving a value of *θ*_*C*_ = 1 is unattainable in reality because some domestication selection is inevitable, we include it as a point of comparison and to provide the complete range; where on this continuum, a given cultured population might sit will depend on a complex combination of broodstock origin, time in captivity, and strength of domestication selection.

## Results

### Degree of maladaptation

**Figure 2:**
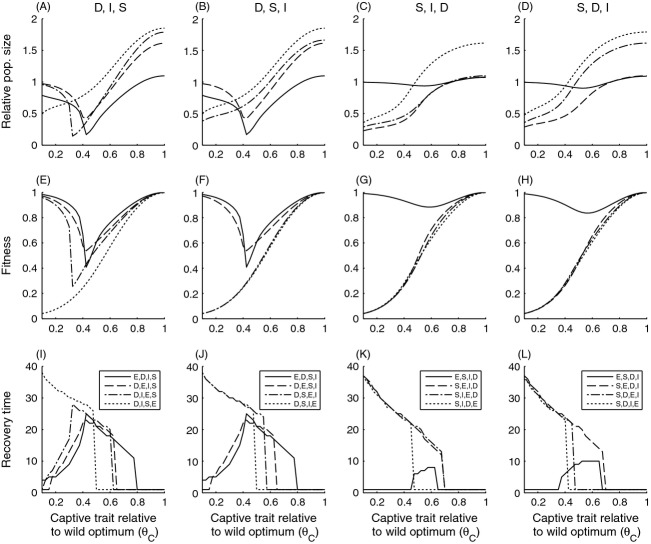
The effect of escape timing and life cycle order: each column has a different sequence of events (D: density dependence, S: selection, I: density-independent mortality), and each line indicates a different escape time (E) within that sequence. We omit all orderings with density-independent mortality immediately following reproduction, as such simulations are mathematically equivalent to reducing the reproductive output *R*, to which we explore sensitivity. Here and in the remaining figures, population size (the first row) and fitness (the second row) are plotted relative to their respective baseline values without the cultured population, and recovery time (the third row) is in generations. For an indication of absolute values of population size and fitness, see Fig. A.1 in Appendix A.

The potential consequences of increasing maladaptation in the aquaculture environment depend on the relative timing of natural selection, escape, and reproduction in the life cycle (Fig. 2). If escape occurs after natural selection and before reproduction, then demographic, fitness, and recovery time consequences of spillover increase with decreasing *θ*_*C*_ (increasing degree of maladaptation in the cultured population). If escape occurs before natural selection, an intermediate minimum in fitness and population size, and an intermediate maximum in recovery time, can occur at intermediate *θ*_*C*_. In this case, selection can purge extremely maladapted cultured-origin individuals (low *θ*_*C*_) before they reproduce and interbreed with wild individuals. In such cases where an intermediate degree of maladaptation has most serious consequences, the effect of the intermediate escapees is greater when density dependence occurs before selection (i.e., hard selection; Fig. 2 first and second columns) than after selection (i.e., soft selection; Fig. 2 third and fourth columns). This result occurs because, under soft selection, selection can remove maladapted cultured escapees before they affect the density-dependent mortality of wild individuals.

### Constant versus pulsed spillover

**Figure 3:**
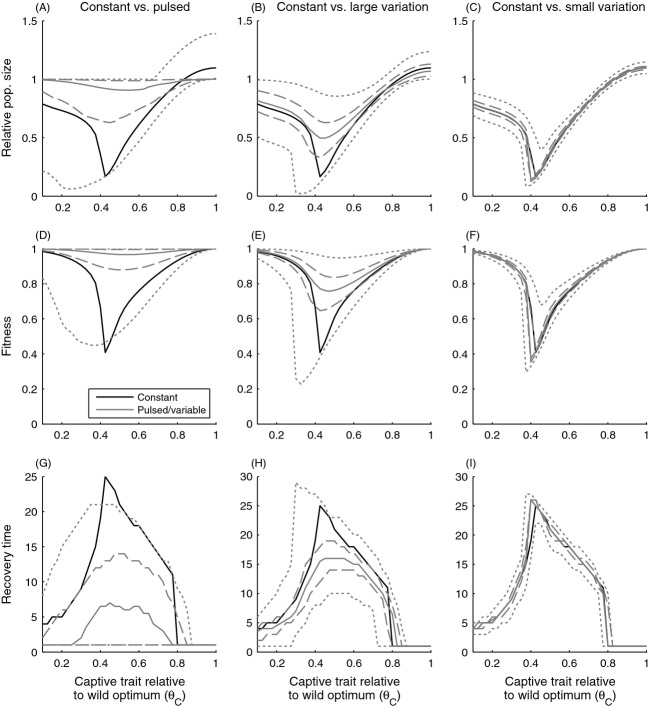
Constant versus pulsed spillover: black lines indicate the outcome given constant, low-level spillover while gray lines give the outcome with stochastically variable spillover. For the variable spillover, the solid lines indicate the median outcome, dashed lines the 25th and 75th percentiles, and dotted lines the 1st and 99th percentiles of the data over multiple runs and generations (see Fig. 4 for example runs). The first column (panels A, D, G) indicates the pulsed case of either entire net-pen escape or no escapees, the second (panels B, E, H) and third (panels C, F, I) columns indicate the variable case with escapees drawn from a binomial distribution with 10 or 100 Bernoulli trials, respectively. For an explanation of *y*-axis values, see Figure 2.

**Figure 4:**
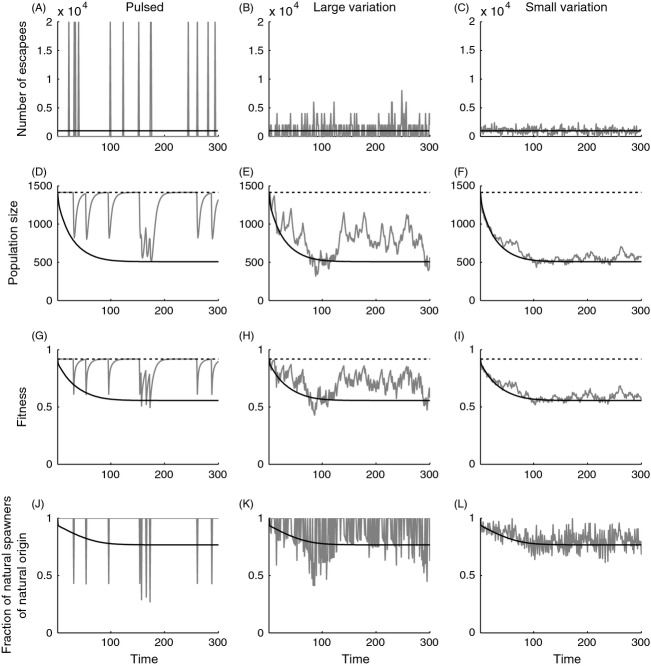
Example time series given constant, pulsed, or variable spillover (with *θ*_*C*_ = 0.6). The first column shows pulsed escapes, and the second and third columns show stochastic escape with large and small variation around a constant mean, respectively. In each plot, the gray lines show the results for the pulsed/variable spillover and the black lines show the constant spillover. In the population size and fitness plots (second and third rows), the broken line indicates the baseline value without aquaculture. The fraction of natural spawners of natural origin (Waples et al. 2012) in the bottom row is calculated as 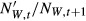 from the values in eqn 7.

Assuming the same average number of escapes over time, pulsed and highly variable spillover have a smaller demographic, fitness, and recovery effect on average than constant spillover (Fig. 3, first and second columns). In test simulation runs, we found that the greater average effect of constant spillover holds for stronger selection, lower levels of spillover on average, and spillover occurring after rather than before density dependence. As the amount of variation in the stochastic spillover decreases (i.e., spillover occurs more frequently but in smaller amounts), the distribution converges toward the constant spillover case (Fig. 3, third column). In other words, increasing the variability in escapes decreases their effect as long as the same average number occur over time. Evaluation of a sample time series indicates that the immediate effect of a stochastic release is a large drop in fitness and population size, followed by a relatively rapid return to natural baseline fitness and population size due to strong selection (Fig. 4). Therefore, between escape events, the population typically returns close to its original value except in rare cases of repeated large escapes occurring sequentially by chance. Under constant, low-level spillover, the population continually experiences weaker, slower effective selection rather than episodes of strong, rapidly effective selection (illustrated using the breeder's equation in Box 1 and Supporting Information Appendix B).

**Box 1: Breeder's equation illustration of selection efficiency**

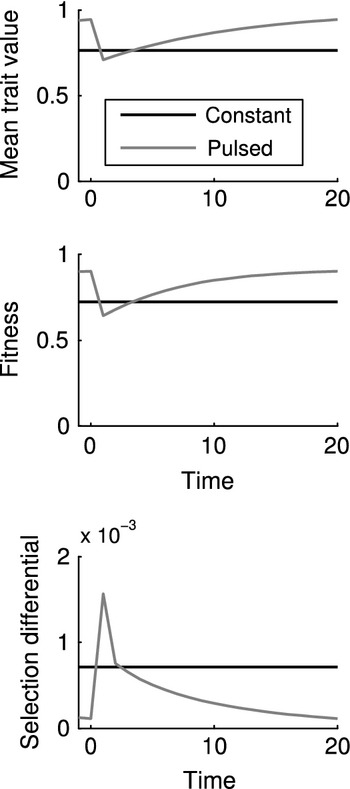

To illustrate the difference in the efficiency in selection in the pulsed spillover versus constant spillover simulations, here, we simplify the model into a form than can be expressed as the breeder's equation, which contains a direct measure of selection efficiency in the selection differential. Specifically, the breeder's equation *R* = *h*^2^*S* expresses the response to selection (change in mean trait) *R* as the product of the trait heritability *h*^2^ and the selection differential *S*. Under the assumptions of a normal distribution with constant phenotypic variation for the trait under selection (which will underestimate the negative fitness effects of maladapted escapees through an increase in genetic variance) and stabilizing selection, we can derive a mathematical expression for the selection differential as it depends on the optimal trait, variance in selection, genetic and environmental variances, mean trait in captivity, and number of escapees at a given point in time (derivation in Supporting Information Appendix B). Under pulsed spillover, the substantial reduction in mean trait and fitness results in a temporary spike in selection strength, which leads to an initially rapid, then slowing, return to the optimal value. Under constant spillover, selection against the smaller number of escapees remains weak through time at equilibrium.

### Sterilization

**Figure 5:**
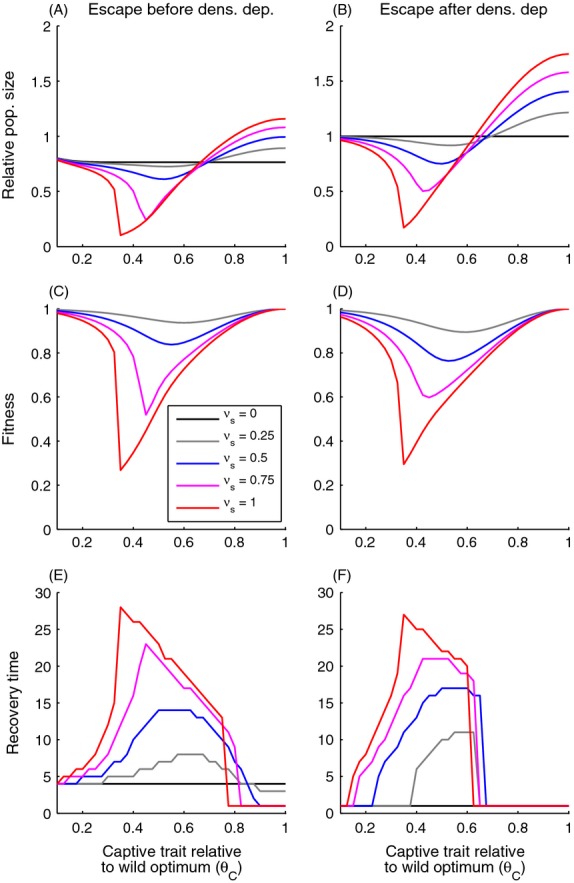
The effect of different values for the relative spawning success of aquaculture origin individuals, where ν_*S*_ = 0 indicates complete sterilization and 0 < ν_*S*_ < 1 can indicate the effect of imperfect sterilization. For an explanation of *y*-axis values, see Fig. 2.

Analysis of the relative spawning success of aquaculture escapees (ν_*S*_) allows us to separate the demographic effect of escapees increasing the density-dependent mortality of wild individuals (population size when ν_*S*_ = 0, or no cultured-origin fish spawn) from the fitness effect that occurs when spawning of cultured-origin individuals and interbreeding with the wild population occurs (Fig. 5). When the cultured-origin individuals are maladapted enough to substantially reduce wild fitness (*θ*_*C*_<∼ 0.5, Fig. 5B), the fitness effects of aquaculture escapees can cause a decline in population size (Fig. 5A) and increase in recovery time (Fig. 5C) as much or greater than that due to their demographic effect. This effect declines rapidly and nonlinearly with decreasing spawning success of cultured-origin individuals, which could represent increasingly effective sterilization or reduced reproductive success due to nongenetic effects of rearing in the cultured environment. In other words, sterilization of 50% of cultured escapes can result in a substantially >50% reduction in fitness consequences (Fig. 5, blue lines).

### Stage of escape

Assuming the same number of escapees regardless of stage, as spillover occurs later in the life cycle, it tends to have greater fitness effects (Fig. 2 middle row). If the cultured population is substantially maladapted to wild conditions (small *θ*_*C*_), then these larger fitness effects translate into larger demographic effects (smaller wild population size in Fig. 2 top row). Alternately, if the cultured population is relatively well adapted to wild conditions (*θ*_*C*_ close to 1), then more escapees survive natural selection, and the total spawner population size is larger (Fig. 2 top row).

### Parameter sensitivity analysis

**Figure 6:**
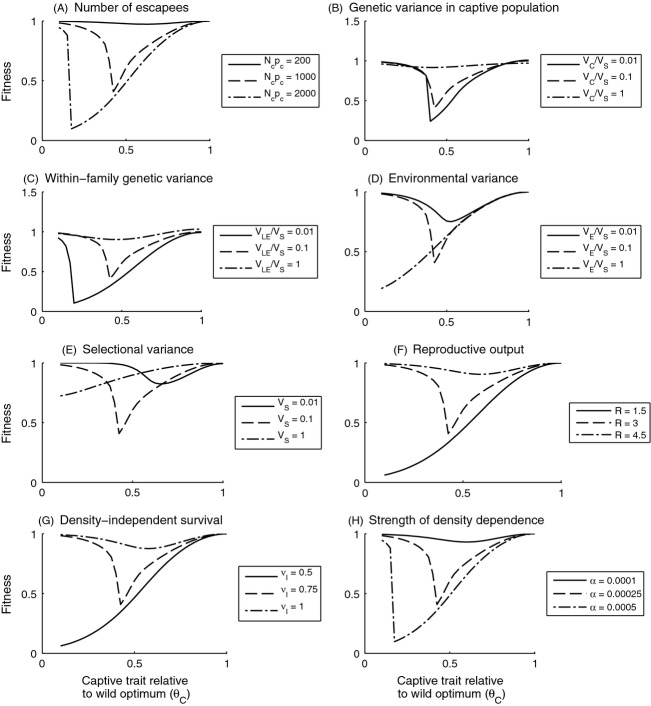
Effect of different parameter values on fitness, plotted relative to the baseline fitness in equivalent simulations without aquaculture escapees. The broken lines indicates the outcome under default parameter values (Table 1), with the solid and dash-dotted lines indicating the effect of decreasing or increasing the value of the indicated parameter, while all others are held constant. Values for the variances in panels (B–D) are expressed relative to the value of the variance in the selection surface *V*_*S*_ (inverse of selection strength). The equivalent plots for population size and recovery time are in Supplementary Information Appendix C.

**Figure 7:**
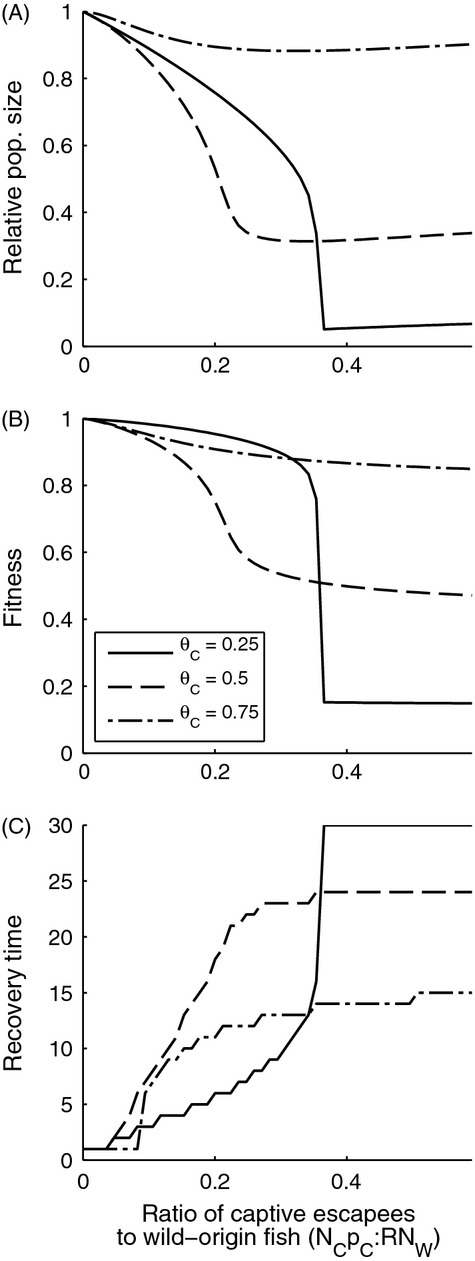
Effect of the ratio of captive escapees to wild-origin fish (per generation) under different values for the mean aquaculture genotype (*θ*_*C*_, relative to the wild optimum phenotype of *θ*_*W*_ = 1). The *x*-axis value of captive-origin:wild-origin population sizes is measured at escape. For an explanation of *y*-axis values, see Fig. 2.

Intuitively, increasing the number of escapees (whether through increasing *N*_*C*_ or *p*_*c*_, which provide mathematically equivalent results under constant escape; Fig. 6A), decreasing the wild reproductive output (Fig. 6F), decreasing the density-independent survival (Fig. 6G), and increasing the strength of competition (Fig. 6H) all lead to greater effects of aquaculture escapees on wild individuals. This greater decline in fitness is realized through both a deeper fitness trough and a fitness trough shifted to the left, such that a greater degree of maladaptation in the cultured population is necessary for natural selection to effectively purge cultured-origin individuals. The increasing fitness effect arises from a greater ratio of cultivated-origin to wild-origin individuals in a given time step by either increasing the aquaculture escapee population size or decreasing the wild population size. Therefore, this ratio and the degree of maladaptation in the aquaculture population interact to determine when substantial fitness effects of aquaculture escapees on the wild population occur (Fig. 7).

**Figure 8:**
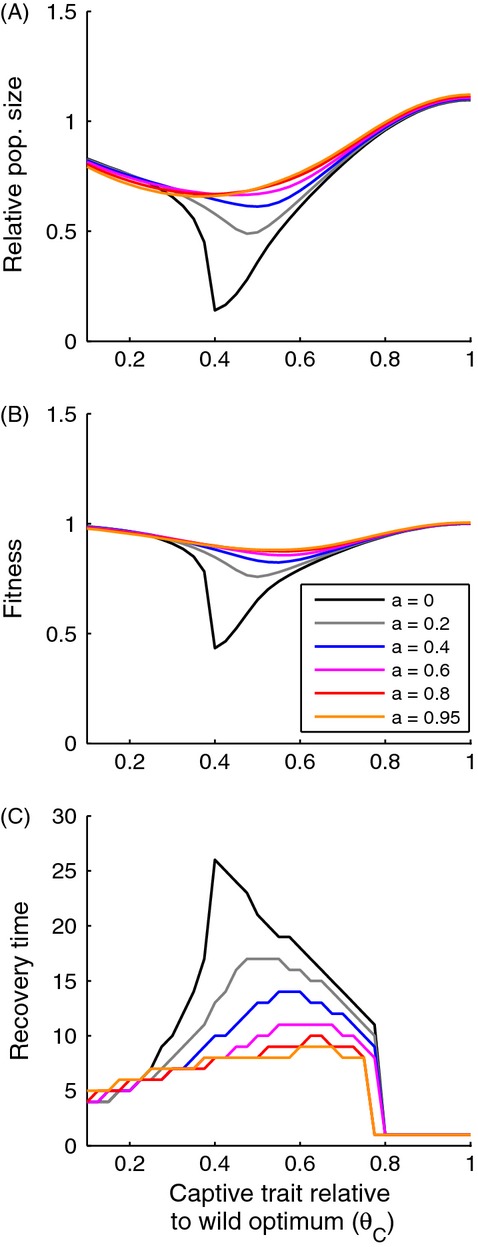
Effect of the strength of assortative mating when included. We implement assortative mating with increasing mating likelihood for increasing phenotypic similarity, where the parameter *a* represents the phenotypic correlation of mating pairs. For an explanation of *y*-axis values, see Fig. 2.

For the additional parameter values, increasing the additive genetic variance in the captive population can model weaker selection in captivity and therefore smaller fitness effects on the wild population (Fig. 6B). Both increasing the within-family genetic variance (Fig. 6C) and decreasing the environmental variance (Fig. 6D) increase the genetic component of the trait under selection, which increases the efficacy of selection and therefore decreases fitness effects of aquaculture escapees. Decreasing the variance in the selection surface (Fig. 6E) increases selection strength and therefore causes a shallower fitness trough at a lower degree of maladaptation in the cultured population (*θ*_*C*_ closer to 1). When incorporating assortative mating such that individuals with more similar phenotypes are more likely to mate with each other (Fig. 8), the fitness effects of maladapted cultured-origin fish decrease because less interbreeding between cultured-origin and wild-origin fish occurs. The demographic and recovery time effects of aquaculture escapees reflect these fitness patterns (Supporting Information Appendix C, Fig. 8).

## Discussion

In our model, as with a variety of existing models of spillover of cultured individuals into wild populations (e.g., Hutchings 1991; Fleming 1995; Tufto 2001; Hindar et al. 2006), both substantial maladaptation in captivity and a large enough number of escapees relative to the wild population size are required to significantly reduce fitness in the wild (Figs 6 and 7). As the ratio of captive escapees to wild-origin fish increases, the fitness effects at first increase slowly, then rapidly, before they plateau to a maximum effect, and the threshold ratio for substantial effects depends on the degree of maladaptation in the cultured population (Fig. 7). This threshold behavior (also noted in a model of spillover from transgenic crops with simpler genetic structure; Haygood et al. 2003) indicates the importance of both the demographic and genetic factors affecting the wild populations. Such effects influence wild population fitness (Fig. 7B), population size (Fig. 7A), and recovery time for small or impacted populations (Fig. 7C; also noted in Hindar et al. 2006). The long recovery times found here suggest the potential for lasting impacts of aquaculture programs that could lead to long phases after aquaculture cessation where reduced wild populations are susceptible to processes such as demographic stochasticity and drift.

Minimization of unintended fitness consequences can therefore depend on both genetic and demographic processes that are under partial management control. Beyond the size of the program, such processes can include the genetic composition of the broodstock, how much spillover is tolerated at different life cycle stages and at what frequency, and to what extent sterilization is implemented. We discuss each of these control options below.

## Degree of maladaptation

Life cycle timing is critical to evaluating the fitness consequences of aquaculture escapes. Specifically, if strong, purifying natural selection occurs between escape and reproduction, an extremely maladapted stock (e.g., nonlocal origin, highly domesticated) could have fitness consequences as low as an aquaculture stock that is only weakly diverged from wild populations (e.g., local origin, recently derived); otherwise, wild population fitness declines monotonically with the degree of maladaptation of the cultured stock (Fig. 2). Here, we show that this result, found in both a model of two-way exchange between hatcheries and cultured environments (Baskett and Waples 2013) and generic models of gene flow across populations experiencing differential selection (Ronce and Kirkpatrick 2001), also applies to the case of one-way migration that arises from aquaculture spillover. The potential for analogous performance of both extremely maladapted and well-adapted cultured populations (also found in the model by Fleming 1995) can help explain why the aquaculture model in Hutchings (1991) did not find a strong role for the fitness difference of cultured individuals from the wild population. Specifically, Hutchings (1991) focused on two extreme values of fitness difference, whereas we find that the sensitivity to fitness difference occurs between these extremes.

Neither an extremely maladapted nor extremely well-adapted cultured population will be easy to achieve in an aquaculture operation. Our results show that minimizing unintended fitness consequences with a strongly domesticated (i.e., extremely maladaptated) broodstock requires either (i) almost 100% effective containment, or (ii) maladaptation extreme enough for near-zero survival in the wild. With respect to the latter, we are not aware of any successful examples of completely avoiding captive wild interbreeding through artificial selection (Naylor et al. 2005). Arriving at such a population through directional selection might require an intermediate phase, with large fitness effects, before all cultured fish are maladapted enough for its success (Thorstad et al. 2008). Conversely, even if the broodstock is initially locally derived, trait differences will inevitably occur because it is impossible to avoid domestication selection in practice (Fleming 1995; Lorenzen et al. 2012) and purposeful selection for desirable traits is economically advantageous (Hutchings and Fraser 2008). Efforts to maintain a diverse set of local cultured stocks will also be costly, although the greater diversity might provide an economic benefit through enhanced viability across stocks (Hutchings and Fraser 2008). The example of a 10-year program in Newfoundland salmon aquaculture to explore the use of local stocks illustrates these challenges: the locally derived stocks had low economic performance and demonstrated genetic differentiation from the wild populations (Pepper et al. 2004).

Therefore, most aquaculture operations will use stocks whose degree of maladaptation is strong enough to produce substantial fitness drag on wild populations, but not so strong that natural selection can effectively purge escaped individuals before they pass on their maladapted genes. The practical consequences of operating in this fitness trough between the two extremes depend on the strength of selection in both the captive and wild environments (where increased variance in Fig. 6B,E reflects decreased selection strength). Stronger natural selection results in a shallower fitness trough with a minimum at lower maladaptation, which suggests that focus captive selection on traits under strong natural selection in the wild will increase the success of using a nonlocal, highly domesticated broodstock (also suggested by the aquaculture model in Tufto 2001). For selection in the captive environment, greater additive genetic variance in the captive population (Fig. 6B) leads to a shallower fitness trough, which suggests that efforts to enhance the genetic diversity in captivity can reduce unintended fitness consequences. An increased ratio of aquaculture escapes to the wild population (whether through increasing the number of escapes in Fig. 6A or decreasing the wild population size through increased density dependence in Fig. 6H) leads to greater consequences at the maladapted extreme (fitness trough shifted to and steeper at lower *θ*_*C*_).

In general, the dependence on the location of the fitness trough on a variety of parameter values (Fig. 6) indicates a strong effect of parameter uncertainty on the consequences of the degree of maladaptation. Exacerbating this parameter uncertainty is model uncertainty such as the genetic architecture underlying the evolving trait (assumed here for simplicity to be many unlinked loci with additive effects), which can significantly impact the evolutionary outcome of the dynamics modeled here (Burke and Arnold 2001; Hansen 2006). Empirical studies of aquaculture–wild hybrids find evidence for both additive (Tymchuk and Devlin 2005; Fraser et al. 2010) and nonadditive (Tymchuk et al. 2007; Normandeau et al. 2009) effects on fitness-related traits. In addition, the depth of the fitness trough will depend on the shape of the relationship between fitness and the trait value (Fleming 1995, here manifest as the shape of the natural selection function). Therefore, a precautionary approach that incorporates containment as well as management of the cultured source population is relevant to cultured populations across the spectrum of potential maladaptation (e.g., broodstock of both local and nonlocal origin).

### The role of assortative mating

The addition to our model of trait-dependent assortative mating, and therefore reduced interbreeding between maladapted cultured escapes and wild fish, substantially decreases fitness effects of cultured escapees (Fig. 8). We find a stronger quantitative effect of assortative mating than in the analogous model of two-way exchange between hatchery and wild populations (Baskett and Waples 2013). The hatchery model incorporated selection by the hatchery during removal of individuals for broodstock, a disruptive selection event that removes exactly those captive-reared individuals with traits that would otherwise make them unlikely to interbreed with wild individuals under assortative mating. Therefore, focusing domestication selection on traits that might influence mating likelihood (e.g., body size, spawn time; Hendry and Day 2005; McLean et al. 2005) is more likely to be effective at reducing fitness consequences in aquaculture (with one-way gene flow) than in hatcheries (with two-way gene flow and disruptive selection that already accomplishes much of what assortative mating would do).

## Constant versus pulsed spillover

We find that the average fitness consequences for wild populations decreases with increasing variability in spillover, assuming the same average number of escapees over time (Fig. 3). This somewhat surprising result, which suggests a greater impact of constant low-level leakage than rare, large pulses of spillover, arises when strong selection rapidly purges maladapted individuals from highly variable spillover (Box 1; in line with the empirical observations of genetic recovery after an individual escape event in Crozier 2000). In comparison, constant spillover has the ratcheting effect of continually decreasing fitness and therefore the wild population size, leading to an increasing proportional makeup of maladapted escapees, further decreasing the overall fitness of the wild population (analogous to the dynamics in migrational meltdown *sensu* Ronce and Kirkpatrick 2001).

Our conclusion here contrasts that of Hindar and Diserud (2007) and Hindar et al. (2006), who suggested a greater effect of large pulses of salmon aquaculture escapees on wild populations. This difference arises because of our focus on equilibrium outcomes as compared to Hindar and colleagues’ emphasis on short-term dynamics. If starting from an unaffected wild population, we can find an initially greater effect of pulsed spillover (Fig. 4) analogous to the shorter-term, transient analyses in Hindar and Diserud (2007) and Hindar et al. (2006), as the ratcheting effect of low-level leakage occurs over many generations. Note that the exact timescale until the effect of low-level leakage occurs is likely to be shorter than modeled here due to a number of dynamics ignored for simplicity and generality. First, multiple traits under selection in the captive environment, while unlikely to affect the equilibrium outcome of models of gene flow (Huisman and Tufto 2012), can affect the rate of evolution from selection in captivity (Araki et al. 2007). Second, some of the traits under selection in captivity (e.g., growth rate; Hutchings and Fraser 2008; Youngson et al. 2001) can lead to shorter generation times. Third, genetic drift can cause spillover effects to occur more rapidly (as occurs in the model of transgenic crops by Haygood et al. 2003). Analogously, our assumption of an additive quantitative genetic trait likely leads to an underestimation of the rate of recovery from a large pulse, as including a limited number of loci and genes of major and minor effects leads to more rapid evolution of individuals in a new environment (here, aquaculture escapes in the natural environment; Gomulkiewicz et al. 2010). Furthermore, adaptive phenotypic plasticity would increase the rate of recovery compared with the random environmental effects assumed here. Such faster recovery would lead to an even smaller long-term effect of rare pulses of spillover in comparison with low-level leakage.

Therefore, a short-term larger effect of rare pulses of spillover, such as from loss of net pens in storms, might mask a longer-term, greater effect of constant, low-level leakage. A crucial driver of this conclusion is our assumption that the same average number of escapees occur over time, but with different levels of variability. In the longest term dataset in the synthesis of Atlantic salmon aquaculture escapes by Morris et al. (2008), this is indeed the case: over the 23 years of data for Magaguadavic River, New Brunswick, an analogous number of farmed salmon adults were detected in total during the many years with low numbers (<250 escapees) and in the two years with high numbers (>700 escapees). More generally, the relative contribution of low-level leakage and large pulses of spillover will depend on the fish being cultured. For example, due to behavioral differences, cod are more likely than salmon to create and escape through holes in aquaculture nets and therefore have greater low-level leakage (Jensen et al. 2010). In addition, management actions taken to reduce large pulses of spillover, such as net-pen reinforcement to reduce likelihood of loss in storms, might reduce a greater number of escapees on average than management actions taken to reduce low-level leakage. Furthermore, our simulations might underestimate the effects of large pulses of spillover by ignoring processes that particularly affect small populations (e.g., genetic drift, inbreeding depression, depensation, demographic stochasticity; Lande 1998), which could occur after a large pulse of escapees, and we do not account for the potential for overcompensatory effects of large pulses. Finally, for simplicity and generality, our explorations assume nonoverlapping generations with one escape event per generation. In reality, for many cultured species such as Atlantic salmon and cod, multiple escape events occur per generation with a high degree of variability in frequency and magnitude across years (Morris et al. 2008), including the potential for multiple ‘pulses’ in a generation. While our ‘large variation’ simulations present a rough first approximation of such scenarios assuming variation in pulse size each generation, a critical next step in the application of these insights to a specific program will be the development of models with overlapping generations to better understand the effect of variability in escape both within and across generations.

Despite these caveats, our results indicate a potential need for greater attention to the role of low-level leakage in driving unintended fitness consequences of aquaculture escapees. Large spillover events typically receive more attention because of the dramatic effect of a large number of escapees at once (Jensen et al. 2010) and they are easier to quantify, such that reliable data on escapees (difficult to obtain in general, Hutchings and Fraser 2008; Jensen et al. 2010; depending on the location, Jackson et al. 2012) are especially lacking for low-level leakage (Thorstad et al. 2008). In other words, the types of escapes that have the greatest impact in our model receive the least monitoring attention. Therefore, while many have suggested the need for improved monitoring efforts on aquaculture spillover (e.g., Hindar et al. 1991; Gross 1998; Naylor et al. 2005; Jensen et al. 2010; Lorenzen et al. 2012), our results add focus to and shift the emphasis of such recommendations by indicating the potential importance of monitoring low-level leakage in particular to quantifying the fitness consequences of aquaculture escapees.

## Sterilization

Sterilization, even if only partially effective, can substantially reduce both genetic and demographic consequences of cultured escapees (Fig. 5). This result, analogous to the importance of hybrid dysgenesis in the aquaculture model by Hutchings (1991), highlights the importance of both demographics and genetics for the effects of aquaculture escapees. Here, the difference between simulations with escape before versus after density-dependent interactions (Fig. 5A,C,E versus Fig. 5B,D,F) quantifies the interaction between demographic and genetic effects. The analogous influence of both genetic and demographic processes is not only evident from these explorations, but also is reflected in the analogous sensitivity of the model outcome to genetic-related parameters (Fig. 6B,C,D,E) and demographic-related parameters (Fig. 6A,F,G,H).

The efficacy of sterilization will clearly depend on the feasibility of management implementation. In particular, sterilization can be economically unattractive if it reduces the performance of aquaculture fish through effects on growth, morphology, and immune system performance (Benfey 1999; Piferrer et al. 2009). Such effects can lead to lower survival of cultured fish in the wild (Thorstad et al. 2008), therefore reducing density-dependent effects and, for partial sterilization, interbreeding more than modeled here. However, sterilization can also lead to reduced reproduction of wild fish due to failed matings between sterilized and wild fish (Hutchings and Fraser 2008; Piferrer et al. 2009), which would lead to greater demographic effects of sterilized fish than modeled here. Therefore, a more refined quantification of the effect of sterilization would depend on improved empirical understanding of the relative influence of both of these processes.

## Stage of escape

We find that later escape typically leads to an increased fitness effects on the wild population (Fig. 2, second row) due to our assumption of the same number of escapees regardless of stage, which then comprise a greater proportion of the total population in each life cycle step subsequent to reproduction. A more realistic parameterization would require empirical data on the number of escapees at different stages, which is typically lacking (Thorstad et al. 2008; but see Morris et al. 2008), and on whether the stage of escape trades off with survival in the wild (e.g., Uglem et al. 2012). A key deviation to the typical trend occurs if cultured escapees affect the density-dependent mortality of the wild population before selection occurs. In this case, the dual demographic and genetic impact of maladapted escapees affecting both the density-dependent survival and fitness of the wild population can lead to a greater maximum fitness effect than if escapees occurred after density dependence but before selection (Fig. 2E,F). Such a dual impact is observed empirically when maladapted farmed escapees and farmed–wild hybrids can competitively displace wild individuals (McGinnity et al. 2003).

Furthermore, escape before density dependence leads to nonlinear effects of parameters or management actions on fitness consequences. For example, the benefits of partially effective sterilization increase more rapidly with increasing efficacy (lower ν_*S*_) when escape occurs before density dependence (Fig. 5 first column versus second column; see also Supplementary Information Appendix D for additional results with escape after density dependence). Because we model a generic trait under selection here, the density-dependent dynamics in our model do not account for the potential for differences in competitive ability between cultured and wild fish (Gross 1998; Youngson et al. 2001; Naylor et al. 2005). Any potential for aquaculture to select for fish with greater competitive ability due to larger body sizes or greater feeding aggression (Gross 1998; Naylor et al. 2005) will increase the demographic effect of aquaculture escapees on wild populations and the importance of the relative timing of escape and density dependence.

The nonlinear effect of escapees through density-dependent interactions also depends on the timing of natural selection. Specifically, early escapees have larger fitness effects on the wild population if they affect density dependence before natural selection (i.e., hard selection, solid lines in Fig. 2E,F) than if they affect natural selection before density dependence (i.e., soft selection, solid lines in Fig. 2G,H). The importance of hard versus soft selection has long been recognized in models of genetic exchange between populations experiencing differential selection (reviewed by Lenormand 2002). In reality, for wild populations affected by cultured fish, natural selection and density dependence occur at a variety of life cycle stages in comparison with the simplified model here (Lorenzen et al. 2012).

## Conclusions

In summary, implications of our model with direct practical relevance to aquaculture planning and management include:

1. All consequences for natural populations scale nonlinearly with the number and viability of escapes (Figs 5 and 7), so prevention of escapes is the most effective way to minimize adverse effects. However, because 100% containment of open net-pen aquaculture is nearly impossible to achieve, other strategies are important to consider.
2. A maladapted (e.g., strongly domesticated, nonlocal broodstock) cultured population can lead to reduced fitness consequences if an episode of strong natural selection occurs after escape and before reproduction; otherwise, fitness consequences increase with increasing maladaptation (Fig. 2). Practical constraints to achieving either an extremely maladapted or extremely well-adapted cultured population mean that most aquaculture operations will operate in the ‘fitness trough’ where escapes can substantially reduce wild population fitness. Predicting the consequences of any particular program will require program-specific data concerning a variety of uncertain demographic and genetic processes such as the strength of selection and strength of density dependence. This factor is critical to evaluating the scale of the project and the trade-offs between potential risks and benefits.
3. Somewhat surprisingly and in contrast to previous analyses of transient dynamics, we find that long-term consequences of steady, low-level escapes are more detrimental than is the case if the same quantity escape in large numbers at rare intervals or otherwise is highly variable in time (Fig. 3). Although minimizing the frequency of very large escape events such as net-pen loss in storms remains crucial, our results suggest that increasing efforts to monitor and prevent constant, low-level leakage from aquaculture operations deserves analogous consideration.
4. Sterilization and other management actions that reduce the number of reproductively viable escapes can substantially reduce consequences of escapes even if they are not 100% successful (Fig. 5). In particular, if containment is effective in restricting most escapes to early life stages before density-dependent mortality occurs in the wild, the benefits can be nonlinear (e.g., if sterilization is only 50% effective, potential reductions in fitness consequences are greater than 50% in Fig. 5c).

While the conclusions with respect to containment and sterilization are expected, quantifying their efficacy, even when imperfectly achieved, here allows comparison with the effects of the degree of maladaptation in the aquaculture population and variation in escape over time for an integrated, comprehensive analysis of a variety of possible management approaches to minimizing unintended fitness consequences. In addition to addressing a variety of the assumptions highlighted elsewhere in the Discussion (e.g., incorporating different genetic architectures, multiple traits under selection, and overlapping generations), future modeling efforts to integrate the fitness effects of escapees explored here with additional effects of aquaculture on wild populations, such as concentrated nutrient release and disease spread (Naylor et al. 2005; Lorenzen et al. 2012), can help to refine the weighting of alternative management options that might coincide or trade-off in their potential to minimize different impacts. Looking beyond aquaculture to additional artificial propagation programs with the potential for interbreeding with wild conspecifics (Laikre et al. 2010), our results indicate the central importance of understanding the relative timing of events within life cycles and the variability of spillover across time to the effective management of unintended fitness consequences.

## Data archiving statement

As this is a modeling study, we did not collect raw data.

## Supporting Information

**Appendix S1.** Assessing strategies to minimize unintended fitness consequences of aquaculture on wild populations.
