# Supplementary material for: Assessing strategies to minimize unintended fitness consequences of aquaculture on wild populations
Source: Evol Appl. 2013 Oct 9;6(7):1090–108. doi: 10.1111/eva.12089 (PMC3804241; doi:10.1111/eva.12089)
Supplement: Supplementary file 1 [file eva0006-1090-SD1.pdf]

Supporting Information for

Assessing strategies to minimize unintended fitness consequences of aquaculture on wild populations

A Full suite of metrics

A number of metrics can describe the genetic and demographic effects of escapees from cultivated populations on wild populations. In the main text we focus primarily on population size, fitness, and recovery time after cessation of an aquaculture program. Here we demonstrate that these metrics capture a wide variety of additional possibilities, with all metrics illustrated in the default case in fig. A.1. In addition, we test the sensitivity of our metrics to the run time chosen.

Two possible metrics of demographic effects of aquaculture are the equilibrium population size (fig. A.1a) and the fraction of natural spawners of natural origin ($N'_{W,t}/N_{W,t+1}$ in eq. 7; fig. A.1g). Both show the maximum negative effect of aquaculture at the same value for the captive mean (θ_C) relative to the wild optimum. However, these metrics have opposing outcomes in comparing the extremes of similar versus different captive populations relative to the wild (θ_C close to 1 versus 0, respectively). For more similar captive populations, more captive individuals survive natural selection, benefiting the wild population size and reducing the fraction of natural spawners of natural origin. For more different captive populations, captive individuals negatively affect the wild population through density-dependent mortality but then are less likely to survive natural selection, thus reducing the wild population size but resulting in a larger fraction of natural spawners being of natural origin.

For genetic effects of aquaculture, fitness (fig. A.1b, black solid line) is integrated over the full breeding value distribution (eq. 6) and thus captures aquaculture effects on both the genetic mean ($\bar{\mu}_W = \int g\bar{\phi}(g)dg$; fig. A.1c) and variance ($\bar{G}_W = \int (g - \bar{\mu}_W)^2\bar{\phi}(g)dg$; fig. A.1d). Note that, as genetic variance changes with the evolution of the full breeding value distribution, heritability ($h^2 = \bar{G}_W/(\bar{G}_W + E)$) changes as well (fig. A.1d, broken grey line). Also, without aquaculture (baseline value indicated by the dotted line in fig. A.1b), fitness is less than unity because of variance load. Fitness ($\bar{W}$) is the direct inverse of migration load (fig. A.1b, broken grey line), calculated as $L = (\bar{W}_b - \bar{W})/\bar{W}_b$ given the equilibrium baseline fitness without aquaculture $\bar{W}_b$ (**?**).

For the effect of aquaculture on the recovery of an impacted or endangered species, in the main text we focus on how long a wild population takes to recover after cessation of an aquaculture program (fig. A.1e). A potentially relevant recovery question in the context of wild endangered species is whether the continued presence of an aquaculture program, with spillover, affects the recovery timeline for the wild population. We measure this potential recovery effect by the time it takes a small wild population (initializing the wild population size at $N_{W,0} = 25$ individuals) to recover to a threshold population size (defined as half of what the wild population would be at equilibrium without aquaculture) given aquaculture escapees (fig. A.1f). Both recovery time metrics follow the same qualitative relationship with θ_C, where the values of θ_C that maximize recovery time without aquaculture lead to a wild population that remains below the threshold ("recovered") population size given continued aquaculture.

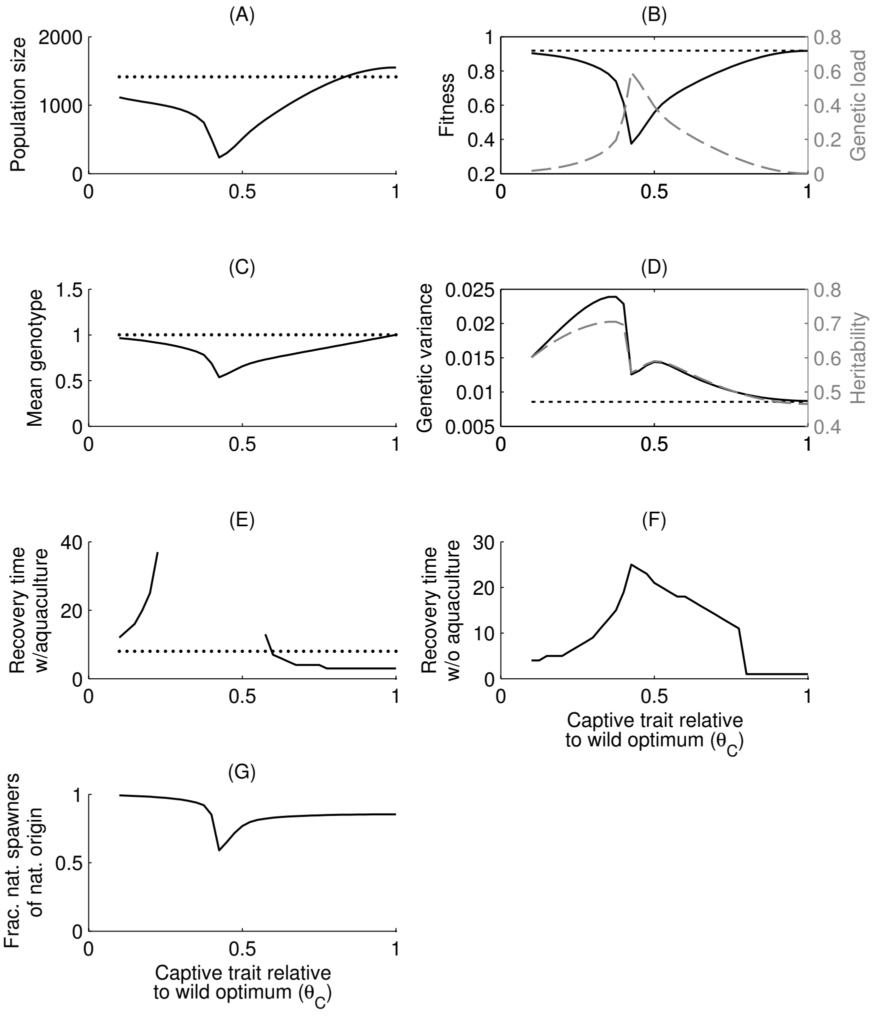

Figure A.1: Equilibrium demographic and genetic metrics as a function of the captive mean trait θ_C under default parameter values and assumptions. Dotted lines indicate baseline values for a wild population without aquaculture. In panel (e), missing data indicates cases where the wild population cannot reach 50% of its natural size given ongoing aquaculture.

Finally, in investigating the three central metrics with different model run times, we find that run time affects the quantitative outcome but not the qualitative trends (fig. A.2). We also confirmed that the same comparative, qualitative trends across model runs as in figs. 1-3 and 5-7 in the main text occurred with a run time of $t_f = 30$ time steps (time to $\sim 50\%$ equilibrium in the default model scenario; results not shown).

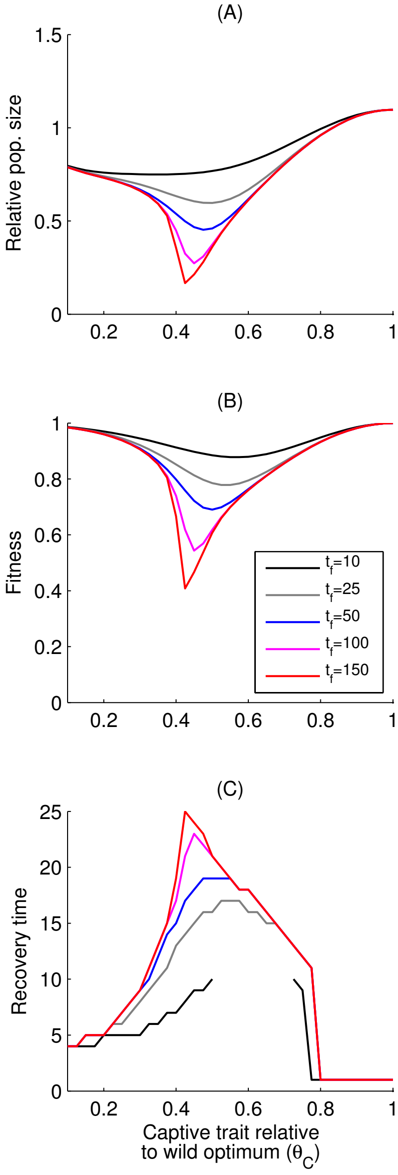

Figure A.2: Model outcome sensitivity to different run times (final time t_f) for the three focal metrics, population size (relative to that without aquaculture), fitness, and recovery time (after cessation of an aquaculture program), under the default life cycle ordering and parameter values.

B Breeder's equation simplification of the model

In order to better understand the dynamics of the strength of selection during the pulsed versus constant spillover simulations, we simplify the model to the analogous breeder's equation version. While providing a tractable metric of dynamical selection efficiency, this approach assumes a normal breeding value distribution with constant genetic variance and therefore underestimates the potential for aquaculture inputs to reduce fitness through increases in genetic variance. In the breeder's equation, the response to selection R is the product of the heritability h^2 and the selection differential S:

$$R = h^2 S. \tag{B.1}$$

The response to selection is the change in mean phenotype $\bar{z}_t$ at time t, i.e., $R = \Delta\bar{z}_t = \bar{z}_t' - \bar{z}_t$. The heritability is the genetic proportion of the total phenotypic variance σ_z^2, which, assuming no genotype-by-environment interaction, is the sum of the additive genetic variance σ_g^2 and environmental variance σ_e^2, i.e., $h^2 = \sigma_g^2/\sigma_z^2$ where $\sigma_z^2 = \sigma_g^2 + \sigma_e^2$. The selection differential is the product of the phenotypic variance and the selection gradient $\beta(\bar{z}_t) = \frac{1}{\bar{W}(\bar{z}_t)}\frac{\partial\bar{W}(\bar{z}_t)}{\partial\bar{z}_t}$ given mean fitness $\bar{W}(\bar{z}_t)$, i.e., $S = \sigma_z^2\beta(\bar{z}_t)$. Therefore, we can re-express eq. B.1 as

$$\bar{z}_t' - \bar{z}_t = \sigma_g^2 \frac{1}{\bar{W}(\bar{z}_t)}\frac{\partial\bar{W}(\bar{z}_t)}{\partial\bar{z}_t} \tag{B.2}$$

(?).

The mean fitness $\bar{W}(\bar{z}_t)$ is the product of the fitness $W(z)$ of each phenotype z and the phenotypic probability distribution $\phi_t(z)$ integrated over all phenotypes, i.e., $\bar{W}(\bar{z}_t) = \int W(z)\phi_t(z)dz$. Given a normally-distributed phenotype distribution with mean $\bar{z}_t$ and variance σ_z^2,

$$\phi_t(z) = \frac{1}{\sqrt{2\pi\sigma_z^2}} e^{-\frac{(z-\bar{z}_t)^2}{2\sigma_z^2}}, \tag{B.3}$$

and a fitness function representative of stabilizing selection for optimal trait θ with variance in the selection curve σ_w^2,

$$W(z) = e^{-\frac{(z-\theta)^2}{2\sigma_w^2}}, \tag{B.4}$$

then the mean fitness is

$$\bar{W}(\bar{z}_t) = \sqrt{\frac{\sigma_w^2}{\sigma_g^2 + \sigma_e^2 + \sigma_w^2}} e^{-\frac{(\bar{z}_t-\theta)^2}{2(\sigma_g^2+\sigma_e^2+\sigma_w^2)}}. \tag{B.5}$$

Using this function, the selection gradient and mean phenotype iteration are

$$\beta(\bar{z}_t) = \frac{\theta - \bar{z}_t}{\sigma_g^2 + \sigma_e^2 + \sigma_w^2} \tag{B.6}$$

$$\bar{z}_t' = \bar{z}_t + \frac{\sigma_g^2(\theta - \bar{z}_t)}{\sigma_g^2 + \sigma_e^2 + \sigma_w^2}. \tag{B.7}$$

To apply the above dynamics to a population with aquaculture input, we first take separately the post-selection $\bar{z}_t'$ (eq. B.7) for each of the wild (with mean phenotype $\bar{z}_t$)

and aquaculture (with mean phenotype $\bar{z}_A$) populations. We then calculate the average phenotype of the combined population, weighted by relative input. This relative input is the post-selection size of each population, where we assume the mean fitness in eq. B.5 provides the origin-dependent survival. Given a reproductive output of $\hat{R}$ and population size at time t of N_t, the wild contribution is $\bar{W}(\bar{z}_t)\hat{R}N_t$. Given a total aquaculture population size of N_A and proportion release at time t of p_t, the aquaculture contribution is $\bar{W}(\bar{z}_A)p_tN_A$. Then the new mean phenotype is

$$\bar{z}_{t+1} = \frac{\bar{W}(\bar{z}_t)\hat{R}N_t\left(\bar{z}_t + \frac{\sigma_g^2(\theta-\bar{z}_t)}{\sigma_g^2+\sigma_e^2+\sigma_w^2}\right) + \bar{W}(\bar{z}_A)p_tN_A\left(\bar{z}_A + \frac{\sigma_g^2(\theta-\bar{z}_A)}{\sigma_g^2+\sigma_e^2+\sigma_w^2}\right)}{\bar{W}(\bar{z}_t)\hat{R}N_t + \bar{W}(\bar{z}_A)p_tN_A}. \tag{B.8}$$

When following population size, we apply a maximum limit K such that

$$N_{t+1} = \min\left(K, \bar{W}(\bar{z}_t)RN_t + \bar{W}(\bar{z}_A)p_tN_A\right). \tag{B.9}$$

This formulation assumes a life cycle ordering of reproduction—escape—selection—density dependence.

In order to determine selection efficiency at each point in time, we use the average selection differential ($\bar{S}_t = \sigma_z^2\beta(\bar{z}_t)$ given the selection gradient in eq. B.6) weighted by the relative contribution of each of the wild and aquaculture populations:

$$\bar{S}_t = (\sigma_g^2 + \sigma_e^2)\left(\frac{\bar{W}(\bar{z}_t)RN_t\beta(\bar{z}_t) + \bar{W}(\bar{z}_A)p_tN_A\beta(\bar{z}_A)}{\bar{W}(\bar{z}_t)RN_t + \bar{W}(\bar{z}_A)p_tN_A}\right). \tag{B.10}$$

To construct the figure presented in Box 1, we numerically iterate this model using analogous parameter values to those in the full model simulations from table 1: $\theta = \theta_W$, $\sigma_w^2 = V_S$, $\sigma_g^2 = V_{LE}$, $\sigma_e^2 = V_E$, $\hat{R} = \nu_I R$, $K = (\hat{R}-1)/(\alpha\hat{R})$, $\bar{z}_A = \theta_C$, $N_C = N_C$, and $p_C = 0.05$ in the constant-spillover simulations or, in the pulsed-spillover simulations, $p_C = 1$ if the year is a multiple of twenty (0.05^{-1}) and 0 otherwise.

C Additional sensitivity results

Here we present the sensitivity of equilibrium population size and recovery time to the same parameter ranges explored in the fitness plot in the main text (fig. 6).

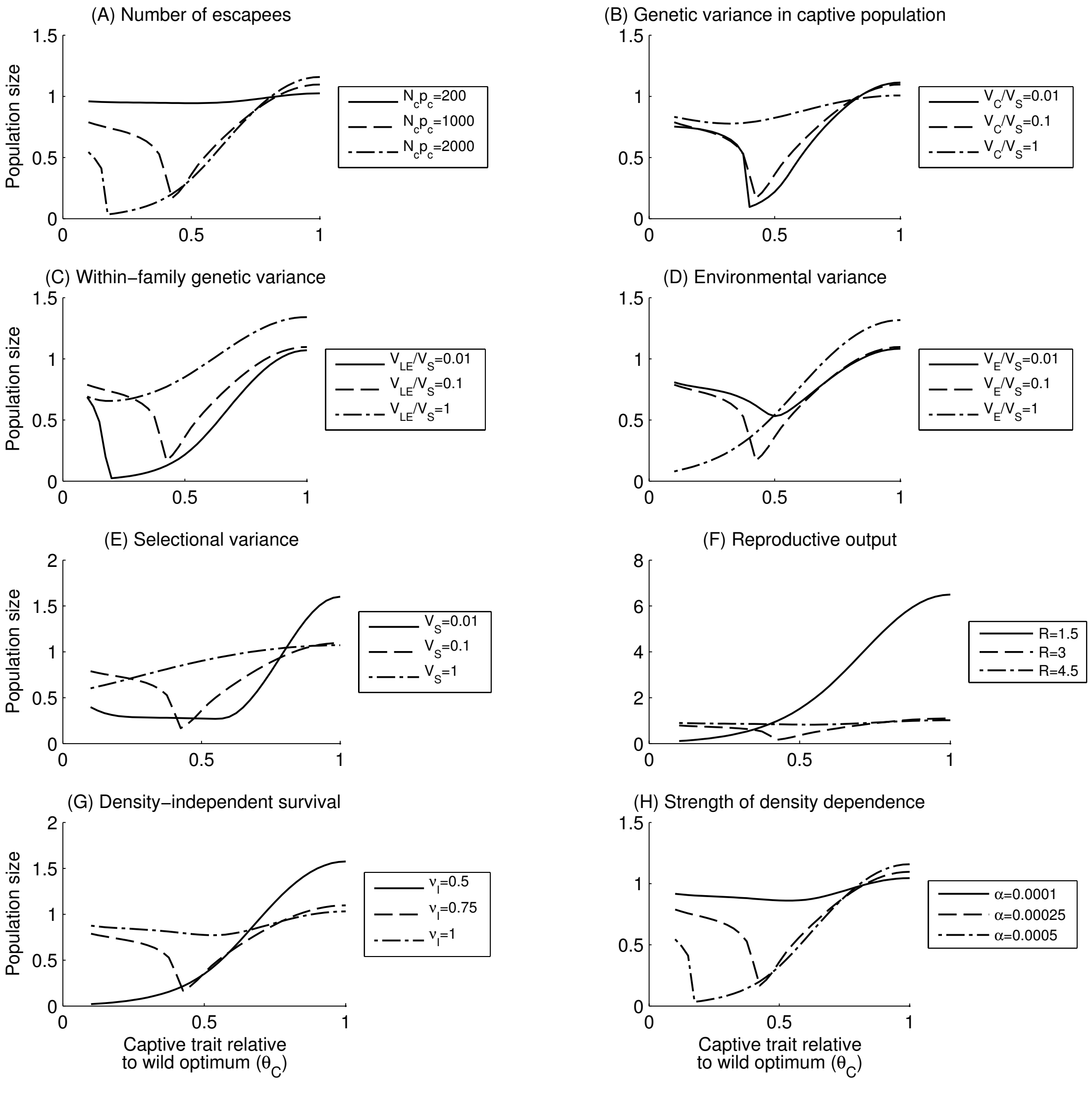

Figure C.1: Effect of different parameter values on population size, plotted relative to the baseline value without an aquaculture population. See fig. 6 for additional details on model implementation. Note differences in y-axis ranges.

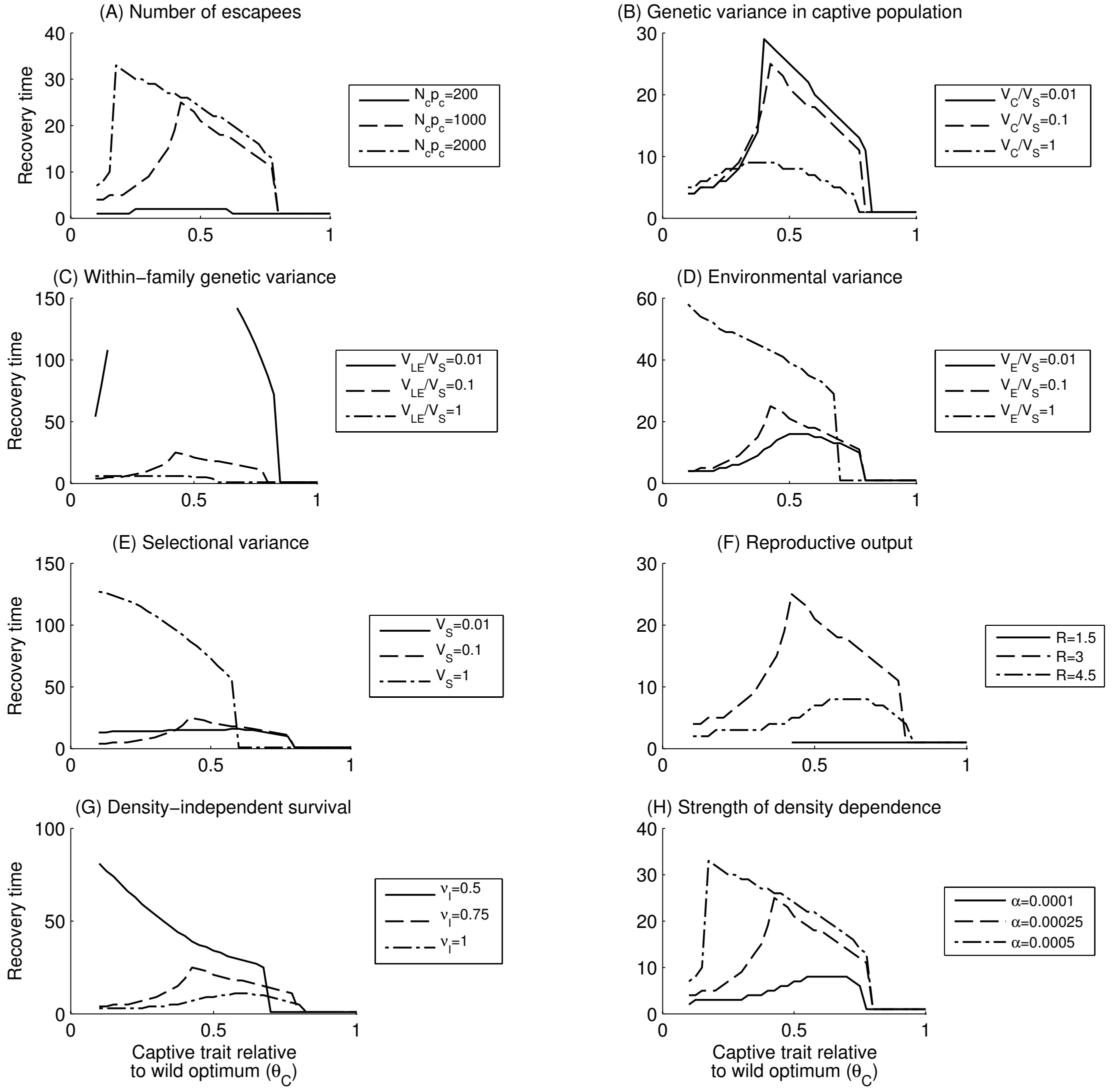

Figure C.2: Effect of different parameter values on time to recovery. See fig. 6 for additional details on model implementation. Note differences in y-axis ranges.

D Additional results with escapement after density dependence

Here we present simulations equivalent to figs. 3 (constant versus pulsed escapement), 7 (ratio of captive escapees to wild-origin fish), and 8 (assortative mating) with escape occurring after density-dependent mortality, as opposed to before (the default assumption in the main text).

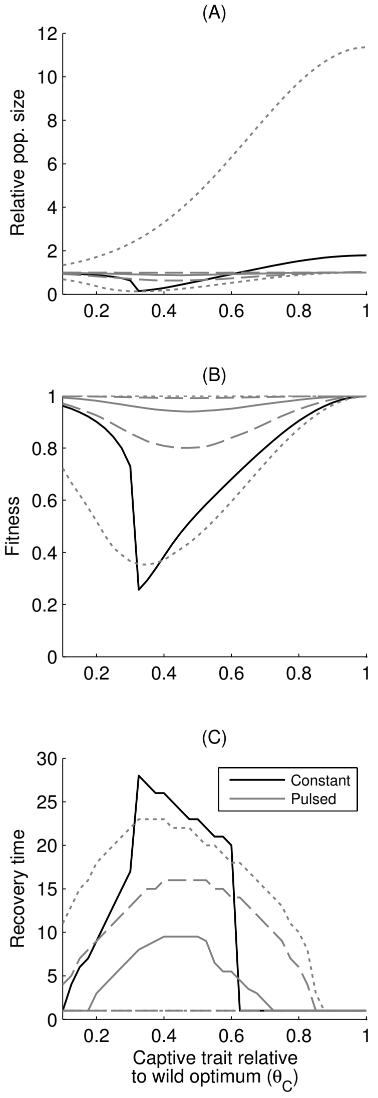

Figure D.1: Constant versus pulsed spillover: equivalent to fig. 3, first column, with escape after density dependence. Black lines indicate the outcome given constant, low-level spillover while gray lines give the outcome with stochastically variable spillover. For the pulsed spillover, the solid lines indicate the median outcome, dashed lines the 25th and 75th percentiles, and dotted lines the 1st and 99th percentiles of the data over multiple runs and years.

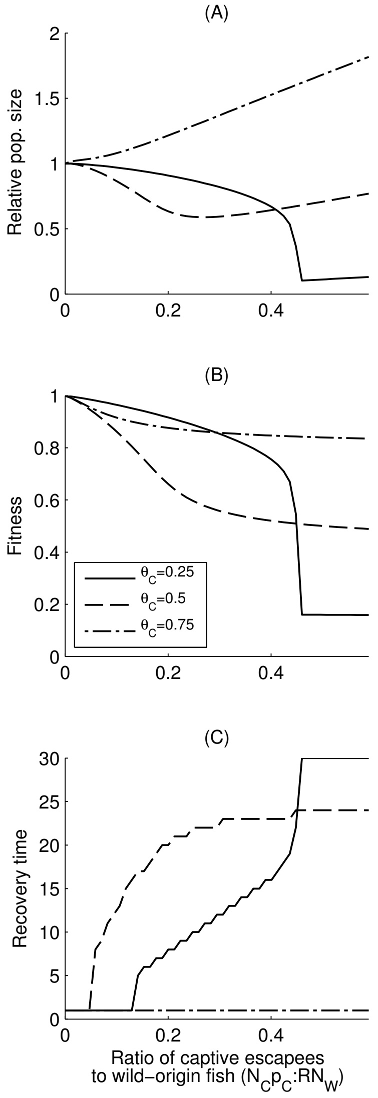

Figure D.2: Effect of the ratio of captive escapees to wild-origin fish: equivalent to fig. 7 with escape after density dependence. Different lines indicate simulations with different values for the mean aquaculture genotype (θ_C, relative to the wild optimum phenotype of $\theta_W = 1$). The x-axis value of captive-origin:wild-origin population sizes is measured at escape.

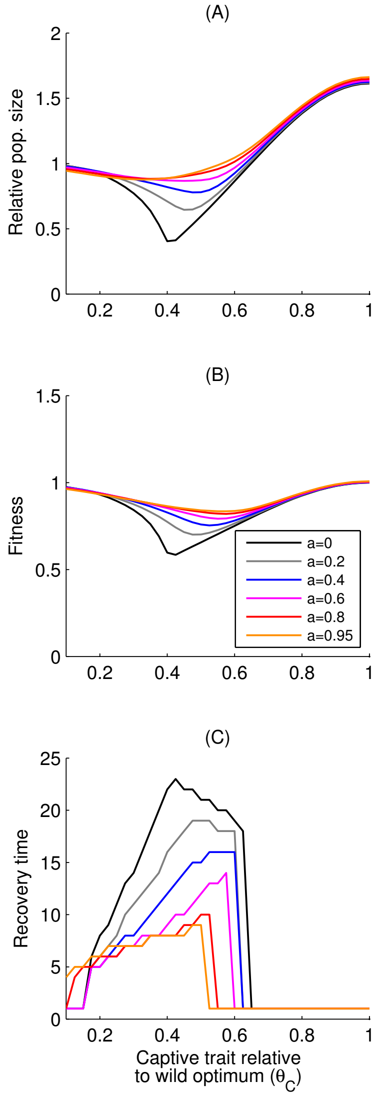

Figure D.3: Effect of assortative mating: equivalent to fig. 8 with escape after density dependence. We implement assortative mating with increasing mating likelihood for increasing phenotypic similarity, where the parameter a represents the phenotypic correlation of mating pairs.
